# Identification of in vivo phosphorylation sites of lens proteins from porcine eye lenses by a gel-free phosphoproteomics approach

**Published:** 2010-02-24

**Authors:** Shyh-Horng Chiou, Chun-Hao Huang, I-Liang Lee, Yi-Ting Wang, Nai-Yu Liu, Yeou-Guang Tsay, Yu-Ju Chen

**Affiliations:** 1 Graduate Institute of Medicine and Center for Research Resources and Development, Kaohsiung Medical University, Kaohsiung, Taiwan; 2 Institute of Biological Chemistry, Academia Sinica, Taipei, Taiwan; 3 Institute of Biochemical Sciences, National Taiwan University, Taipei; Chemical Biology and Molecular Biophysics Program, Taiwan International Graduate Program, Institute of Chemistry, Academia Sinica, Taipei, Taiwan; 4 Institute of Biochemistry and Molecular Biology, National Yang-Ming University, Taipei, Taiwan

## Abstract

**Purpose:**

Phosphorylation is an important post-translational modification for the cellular regulation of various biosignaling pathways. We have identified in vivo phosphorylation sites of various lens proteins including especially the major structural proteins of the crystallin family from porcine eye lenses by means of two-dimensional gel electrophoresis (2-DE) or immobilized metal affinity chromatography (IMAC) followed by liquid chromatography coupled with tandem mass spectrometry (LC-MS/MS).

**Methods:**

For the identification of phosphorylated residues in various lens proteins of porcine lens extracts, we have adapted two complementary proteomic approaches, i.e., pre-fractionation of protein samples with 2-DE or enrichment of phosphopeptides with IMAC followed by LC-MS/MS analysis and database search. The results were compared and validated with those in phosphoproteomics databases.

**Results:**

Two subunits of α-crystallin, αA-crystallin and αB-crystallin, as well as other lens crystallins and non-crystallin cellular proteins, such as β-enolase, heat shock protein β-1 (HSP27), and glucose-6-phosphate isomerase (GPI) were found to be phosphorylated in vivo at specific sites. Moreover, αA- and αB-crystallins were found to be the most abundantly phosphorylated proteins in porcine lenses, being extensively phosphorylated on serine or threonine, but not on tyrosine residues.

**Conclusions:**

The complementary gel-based and gel-free proteomic strategies have been compared and evaluated for the study of crystallin phosphorylation from whole tissue extracts of porcine eye lenses. Technically, the IMAC method facilitates direct site-specific identification of phosphorylation residues in lens proteins, which does not necessitate the pre-MS/MS 2-DE separation of protein samples. Moreover, the improved strategy using gel-free phosphoproteomics analysis affords a more effective and simplistic method for the determination of in vivo phosphorylation sites than the conventional 2-DE pre-separation of protein mixture. This study should form a firm basis for the comprehensive analysis of post-translational modification of lens proteins in terms of aging or various diseased states.

## Introduction

Mammalian eye lenses are composed of elongated fiber cells, of which approximately 90% of the total soluble proteins belong to three major classes of proteins, i.e., α−, β−, and γ−crystallins [1,2]. Essentially, these crystallins can exist in the eye lens with little turnover throughout the entire lifespan, albeit with various degrees of post-translational modifications such as deamidation, phosphorylation, and proteolytic truncation [3-5]. Among these, phosphorylation is most noteworthy for playing a major role in the regulation of various biosignaling pathways [6] which may include cancer development, aging, and cataract formation. Therefore identification of protein phosphorylation and its exact locations in proteins or enzymes of interest are always considered as a preeminent and nontrivial task in the conventional mechanistic and functional study of various cellular proteins. Mainly attributable to the advent of emerging proteomics, the investigation of protein phosphorylation has recently become less tedious and more amendable to routine analysis [7].

The common strategy of most conventional proteomic approaches to the identification of proteins rests in the peptide mass fingerprints of proteins under study, which can be used as an identification tag to search the corresponding identical or highly homologous sequence fragment patterns in protein sequence databank. Such fingerprints usually come from the tandem mass spectra of peptides generated from proteolytic digestion of proteins of interest. However before obtaining the digested protein fragments, the global or comprehensive separation of a given protein mixture is generally required. 2-DE gel electrophoresis was previously considered as the method of choice, as it could afford a high throughput and relatively high-resolution analytical tool to resolve and separate a mixture of thousands of protein species with different charge and size properties [8]. However, the serious drawback of low sensitivity and under-representation for some special classes of proteins such as the extremely basic or acidic groups of proteins and membrane proteins [8,9] necessitated the development of more sensitive labeling methods such as stable isotopic labeling [10] in conjunction with multidimensional LC-MS/MS analysis. Thus, direct digestion of total cellular protein extracts followed by high-resolution LC-MS/MS, the so-called shotgun strategy, has been shown to facilitate the highly sensitive identification of protein mixtures without prior protein separation on 2-DE gels [7,11,12].

In spite of the rapid improvement of various types of mass spectrometry designed to study post-translational modifications of cellular proteins, especially concerning protein phosphorylation, there still exist some discrepancies or ambiguities between results obtained from previous investigations of different laboratories. The major emphasis of recent proteomic studies is being directed toward a more facile and global analysis of cellular systems, however methodologies to date still do not exist for conducting a routine and reliable high-throughput analysis of proteome-wide changes in the phosphorylation of proteins. In this study, phosphorylated and nonphosphorylated lens proteins from porcine eye lenses were identified by gel-based 2-DE protein fractionation and gel-free enrichment of phosphopeptides from trypsin-digested protein mixture on immobilized metal affinity chromatography (IMAC), followed by LC-MS/MS. Based on our results of the comparison and evaluation of two different protocols of proteomic approaches, we conclude that gel-free IMAC phosphopeptide enrichment, coupled with LC-MS/MS analysis, is now capable of identification of phosphorylated sites from the whole lens extract, effectively circumventing the need for prior protein separation by two-dimensional gel electrophoresis.

## Methods

### Chemicals

Triethylammonium bicarbonate (TEABC) and iron chloride (FeCl_3_) were purchased from Sigma Aldrich (St. Louis, MO). The BCA^TM^ protein assay reagent kit was obtained from Pierce (Rockford, IL). Ammonium persulfate and N, N, N’, N’-tetramethylenediamine were purchased from Amersham Pharmacia (Piscataway, NJ). Acetic acid (AA) was purchased from J. T. Baker (Phillipsburg, NJ). Trifluoroacetic acid (TFA), formic acid (FA), and HPLC-grade acetonitrile were purchased from Sigma Aldrich (St. Louis, MO). Modified, sequencing-grade trypsin was purchased from Promega (Madison, WI).

### Preparation of porcine lens extract

Young porcine eyeballs were obtained from a local slaughterhouse. Eyeballs were kept and stored at −80 °C in a freezer before dissection. Porcine lenses were removed from the eyeballs, homogenized, and suspended in the buffer of 20 mM Tris-HCl, pH 6.8 for the extraction of total lens crystallins as described previously [13-17].

### Two-dimensional gel electrophoresis

Porcine lens extract was solubilized in lysis buffer containing 8 M urea, 0.5% CHAPS or Triton X-100. After the estimation of protein content using a 2-D Quant Kit (Amersham Biosciences, Uppsala, Sweden), about 100 μg total protein was loaded onto IPG gel strips (pH 3–10 Nonlinear, 24 cm, Amersham Biosciences, Uppsala, Sweden). The IPG strips were rehydrated overnight according to the operational guideline of the manufacturer (Amersham Biosciences, Uppsala, Sweden). For the first-dimensional separation, isoelectric focusing (IEF) was performed using Ettan IPGphor II (Amersham Biosciences, Uppsala, Sweden) at 20 °C with 300–8,000 V for 16 h. After IEF, the IPG strips were equilibrated for 10 min each in two equilibration solutions (50 mM Tris-HCl, pH 8.8, 6 M urea, 2% SDS, 30% glycerol containing 100 mg dithiothreitol [DTT] or 250 mg iodoacetic acid [IAA], respectively), and then attached to a 12.5% SDS-polyacrylamide gel of Laemmli’s buffer system, then covered by 0.5% agarose gel. 2-DE was conducted at 130–250 V for 5–6 h until the bromophenol blue reached the bottom of the gel. The gels were stained by Sypro-Ruby overnight. The protein profiles of the gels were scanned using a Typhoon 9400 scanner (Amersham Biosciences, Uppsala, Sweden). Gel image matching was done using ImageMaster^TM^ 2D Platinum Software Version 5.0 (Amersham Biosciences, Uppsala, Sweden). Intensity levels were normalized between gels as a proportion of the total protein intensity detected for the entire gel.

### In-gel digestion

Based on the 2D gel analysis of samples, differentially expressed proteins were selected for further identification by LC-MS/MS. The protein spots were cut from 2D gels, and then destained three times with 25 mM of ammonium bicarbonate buffer (pH 8.0) in 50% acetonitrile (ACN) for 1 h. The gel pieces were dehydrated in 100% ACN for 5 min and then dried for 30 min in a vacuum centrifuge. Enzyme digestion was performed by adding 0.5 μg trypsin in 25 mM of ammonium bicarbonate per sample at 37 °C for 16 h. The peptide fragments were extracted twice with 50 μl 50% ACN/ 0.1% TFA. After removal of ACN and TFA by centrifugation in a vacuum centrifuge, samples were dissolved in 0.1% formic acid as well as 50% ACN.

### LC-MS/MS analysis from 2-DE

Electrospray mass spectrometry was performed using a Finnigan LTQ Orbitrap hybrid mass spectrometer interfaced with Agilent 1200 capillary high-performance liquid chromatography (HPLC) system. A 100×0.075 mm Agilent C18 column (3.5 μm particle diameter) with mobile phases of A (0.1% formic acid in water) and B (0.1% formic acid in acetonitrile) were used. The peptides were eluted at a flow rate of 0.4 μl/min with an acetonitrile gradient, which consisted of 5%–10% B in 5 min, 10%–50% B in 25 min, and 50%–95% B in 4 min. The spectra for the eluting fractions were acquired as successive sets of scan modes. The MS scan determines the intensity of the ions in the m/z range of 200 to 2,000, and a specific ion was selected for a tandem MS/MS scan. The former examined the charge number of the selected ion and the latter acquired the spectrum (CID spectrum or MS/MS spectrum) for the fragment ions derived by collision-induced dissociation. Proteins were identified in NCBI databases by use of MS/MS ion search with the search program Mascot.

### Comprehensive PTM mapping analysis

The data interpretation steps were facilitated by Xcalibur and TurboSequest softwares (Thermo electron, san Jose, CA) as well as in-house proprietary programs. Our Excel macro Output Plus can extract MS and MS/MS data and store them as text files. SegMS macro can generate segmental average MS scans using the above MS data. The macro PTMFinder can use the segmental average MS scan and TurboSequest results to screen the likely modification-containing peptides. For modified candidate peptides with acquired MS/MS spectra, we use another macro MS2Graph to verify their identities along with identification of their modified residues within the peptides for further validation.

### Gel-assisted digestion

The protein samples from the lens were subjected to gel-assisted digestion. The sample was incorporated into a gel directly in the Eppendorf vial with acrylamide/ bisacrylamide solution (40%, v/v, 29:1), 10% (w/v) APS, 100% TEMED as a proportion (14:5:0.7:0.3) [9,18]. The gel was cut into small pieces and washed several times with 25 mM TEABC containing 50% (v/v) ACN. The gel samples were further dehydrated with 100% ACN and completely dried using SpeedVac. Proteolytic digestion was then performed with trypsin (protein:trypsin=50:1, g/g) in 25 mM TEABC with incubation overnight at 37 °C. The tryptic peptides were dried completely under vacuum and stored at −30 °C.

### IMAC Procedure

The IMAC column was first capped at one end with a 0.5 μm frit disk enclosed in a stainless steel column-end fitting. The Ni-NTA resin was extracted from spin column (Qiagen, Hilden, Germany) and packed into a 10 cm microcolumn (500 μm i.d. PEEK column; Upchurch Scientific/ Rheodyne, Oak Harbor, WA) as described previously [19]. Automatic purification of phosphopeptides was performed by connecting to an autosampler and an HP1100 solvent delivery system (Hewlett-Packard, Palo Alto, CA) with a flow rate 13 µl/min. First, the Ni^2+^ ions were removed with 100 µl 50 mM EDTA in 1 M NaCl. Then the IMAC column was activated with 100 µl 0.2 M FeCl_3_ and equilibrated with loading buffer for 30 min before sample loading. The loading buffer/ acetic acid was 6% (v/v) and the pH was adjusted to 3.0 with 0.1 M NaOH (pH=12.8). The peptide samples from trypsin digestion were reconstituted in the loading buffer and loaded into the IMAC column that had been equilibrated with the same loading buffer for 20 min. The unbound peptides were then removed with 100 μl of washing solution, consisting of 75% (v/v) loading buffer and 25% (v/v) ACN, followed by equilibration with loading buffer for 15 min. Finally, the bound peptides were eluted with 100 µl 200 mM NH_4_H_2_PO_4_ (pH 4.4). Eluted peptide samples were dried under vacuum and then reconstituted in 0.1% (v/v) TFA (40 μl) for further desalting and concentration using ZipTips^TM^ (Millipore, Bedford, CA).

### LC-MS/MS analysis from gel-assisted digestion and IMAC

Purified phosphopeptide samples were reconstituted in 4 µl buffer A (0.1% formic acid (FA) in H_2_O) and analyzed by LC-Q-TOF MS (Waters Q-TOF^TM^ Premier; Waters Corp, Milford, MA). For LC-MS/MS analysis by Waters Q-TOF^TM^ Premier, samples were injected into a 2 cm×180 μm capillary trap column and separated by 20 cm×75 μm Waters1 ACQUITYTM 1.7 mm BEH C18 column using a nanoACQUITY Ultra Performance LC^TM^ system (Waters Corp.). The column was maintained at 35 °C and bound peptides were eluted with a linear gradient of 0%–80% buffer B (buffer A, 0.1% FA in H_2_O; buffer B, 0.1% FA in ACN) for 120 min. MS was operated in ESI positive V mode with a resolving power of 10,000. NanoLockSpray source was used for accurate mass measurement and the lock mass channel was sampled every 30 s. The mass spectrometer was calibrated with a synthetic human [Glu^1^]-Fibrinopeptide B solution (1 pmol/µl, from Sigma Aldrich) delivered through the NanoLockSpray source. Data acquisition was operated in the data directed analysis (DDA). The method included a full MS scan (m/z 400–1,600, 0.6 s) and three MS/MS (m/z 100–1,990, 1.2 s each scan) sequentially on the three most intense ions present in the full scan mass spectrum.

### Database search and data-filtering

Raw MS/MS data were converted into peak lists using Distiller (version 2.0; Matrix Science, London, UK) with default parameters. All MS/MS samples were analyzed using Mascot (version 2.2.1; Matrix Science, London, UK). Mascot was set up to search the Swissprot_Mammalia (version 54.2, 55,307 entries) assuming trypsin as the digestion enzyme. Mascot was searched with a fragment ion mass tolerance of 0.1 Da and a parent ion tolerance of 0.1 Da. Two missed cleavages were allowed for trypsin digestion. Phosphorylation (Ser/Thr/Tyr) and oxidation (Met) were selected as variable modifications. To evaluate the false discovery rate of protein identification, we repeated the search using identical search parameters and validation criteria against a randomized decoy database created by Mascot. The false discovery rates with Mascot score >36 (p<0.05) was 0.73% in this study.

## Results and Discussion

The availability of complete genome sequences is moving biologic research to an era where cellular systems are analyzed as a whole rather than as individual components. While global gene expression measurements at the mRNA level opens the door to important biologic advances, much of the understanding of cellular systems and the roles of various cellular constituents still depends on proteomics. The study of proteins at the level of the cellular systems using the current proteomics methodology will provide a firm basis for understanding the complex biosignaling pathways of the whole organism within the interdisciplinary realm of systems biology [20]. Therefore, the global understanding of cellular systems revealed by proteomic investigations will create new avenues of research unlikely to arise from the past paradigm of “single” protein characterization methodologies.

Studies estimate that as many as one-third of all cellular proteins derived from mammalian cells are phosphorylated [6]. Although greater emphasis is being directed toward a comprehensive global analysis of cellular systems, methodologies still do not exist for reliable, high-throughput analysis of proteome-wide changes in the phosphorylation of proteins. Direct determination of individual phosphorylation sites occurring on phosphoproteins in vivo has been difficult to date, typically requiring the purification to homogeneity of the phosphoprotein of interest before analysis. There has been a need for a more rapid and general method for the analysis of protein phosphorylation in complex protein mixtures [21]. In this study, phosphorylated and nonphosphorylated lens proteins from porcine eye lenses were identified and compared by two complementary proteomic protocols, i.e., (1) gel-based 2-DE protein fractionation and (2) gel-free enrichment of phosphopeptides from trypsin-digested protein mixture on immobilized metal affinity chromatography (IMAC) followed by LC-MS/MS. We attempt to evaluate and establish a simplistic protocol to study the post-translational modifications, especially phosphorylation, on the whole lens extract.

Previous reports regarding the investigation of phosphorylation sites of lens crystallins started from the observation that radiolabeled inorganic phosphate (^32^Pi) could be incorporated into both αA- and αB-crystallins with some evidence that serine was the only phosphorylated residue [22]. In vitro phosphorylation of αB-crystallin was later found to be located principally at Ser-45 and Ser-59 [23], in contrast to the in vivo phosphorylated sites at Ser-19 and Ser-45 [24]. For αB-crystallin in the lens, the major phosphorylation sites have been confirmed to be at serine residues 19, 45, and 59 and the phosphorylation at Ser-45 results in uncontrolled aggregation [4,25]. The phosphorylation of αA-crystallins was also identified by mass spectrometry [26]. Thus, there appeared to be some discrepancies or ambiguities, especially concerning different phosphorylated sites of crystallins under in vivo and in vitro conditions between previous investigations in the literature.

### Gel-based proteomic analysis of porcine lens extract

**Figure 1:**
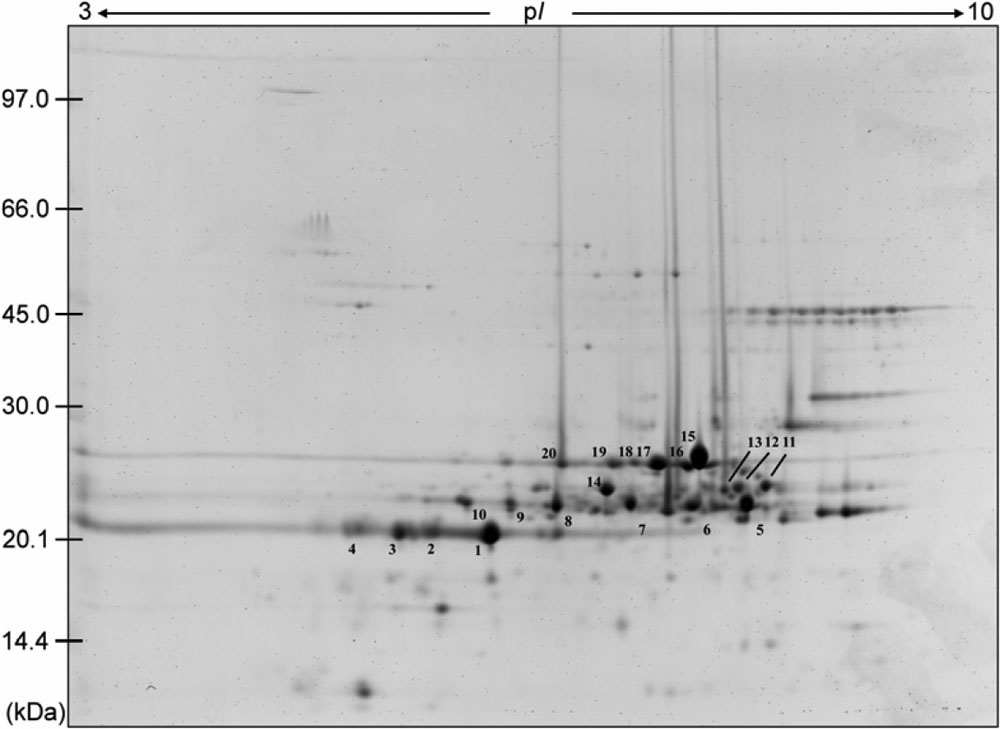
2-DE gel patterns of porcine lens proteins. Total protein (100 μg) in each sample was loaded onto immobilized pH gradient (IPG) gel strips (pH 3–10 Nonlinear, 13 cm). For the first-dimensional separation, IEF was performed using Ettan IPGphor II (Amersham Biosciences) at 300–8,000 V for 16 h. After IEF, the IPG strips were equilibrated in SDS-urea buffer and placed onto the second-dimensional SDS–PAGE. After electrophoresis, the gels were fixed in 10% methanol and 7% acetic acid and stained by Sypro-Ruby. The IPG strips were rehydrated, and after IEF, subjected to 2-DE. Protein spots marked by No. 1–20 on the map were further identified by nano LC-MS/MS and listed in Table 1. It is noteworthy that porcine lenses contain many protein isoforms which present themselves as a series of parallel spots with similar molecular masses in 2-DE profiles. The result is representative of three independent experiments.

The global protein-expression profile of porcine lens was analyzed using high-resolution 2-DE. The p*I* range for the first-dimension IEF strips was 3–10 and the second-dimension SDS–PAGE was run at 12.5% polyacrylamide gel (Figure 1). The proteomic analysis showed that most lens proteins located on the basic and low molecular weight regions correspond to lens crystallins (protein spots No. 1–20) as revealed by LC-MS/MS (Table 1). In our analysis, seven protein spots located on the acidic and basic region on 2D gel were identified as αA-crystallin (protein spots No. 1–4) and αB-crystallin (protein spot No. 5–7), respectively. We also detected 13 protein spots that belong to the class of β-crystallin (protein spots No. 8–20).

**Table 1:** Proteins of porcine eye lens identified by 2-DE and LC-MS/MS analysis.

**Spot**	**Protein name**	**Accession number**	**Mascot Score**	**pI/mass, kDa**	**Normalized spot intensity, %**	**Phosphopeptides**
1	αA-crystallin	P02475	536	5.78/19.7	8.9	
2	αA-crystallin	P02475	428	5.78/19.7	3.0	
3	αA-crystallin	P02475	442	5.78/19.7	3.4	
4	αA-crystallin	P02475	272	5.78/19.7	1.8	
5	αB-crystallin	Q7M2W6	438	6.76/20.1	5.4	RPFFPFHSPSR
6	αB-crystallin	Q7M2W6	465	6.76/20.1	2.8	RPFFPFHSPSR
7	αB-crystallin	Q7M2W6	358	6.76/20.1	2.9	
8	βA4-crystallin	XP_001927427	335	5.91/22.4	2.8	
9	βA4-crystallin	XP_001927427	437	5.91/22.4	1.0	
10	βA4-crystallin	XP_001927427	351	5.91/22.4	0.9	
11	βB3-crystallin	XP_001929508	390	6.36/24.2	1.5	
12	βB3-crystallin	XP_001929508	266	6.36/24.2	1.5	
13	βB2-crystallin	XP_001924958	112	6.45/23.3	0.9	
14	βA2-crystallin	NP_776949	262	6.15/22.2	3.1	
15	βB2-crystallin	XP_001924958	534	6.45/23.3	9.4	
16	βB3-crystallin	XP_001929508	658	6.36/24.2	2.8	
17	βB2 -crystallin	XP_001924958	321	6.45/23.3	5.5	
18	βB2 -crystallin	XP_001924958	390	6.45/23.3	0.9	
19	βB2 -crystallin	XP_001924958	193	6.45/23.3	1.4	
20	βA4 -crystallin	XP_001927427	206	5.91/22.4	1.3	

In the “Phosphopeptides” column, the phosphorylation sites are underlined.

To further characterize whether the proteins in porcine lens were phosphorylated, we enriched the phosphorylated peptides by performing IMAC protocol followed by phosphorylation site identification with LC-MS/MS. As shown in Table 1, the MS/MS data indicated that either Ser-19 or Ser-21 of the peptide RPFFPFHS^19^PS^21^R in αB-crystallin was phosphorylated. However, we could not find any phosphopeptides in trypsin-digested protein spots corresponding to other lens proteins when using the 2-DE approach.

### Gel-based proteomic analysis of phosphorylated αB-crystallin

**Figure 2:**
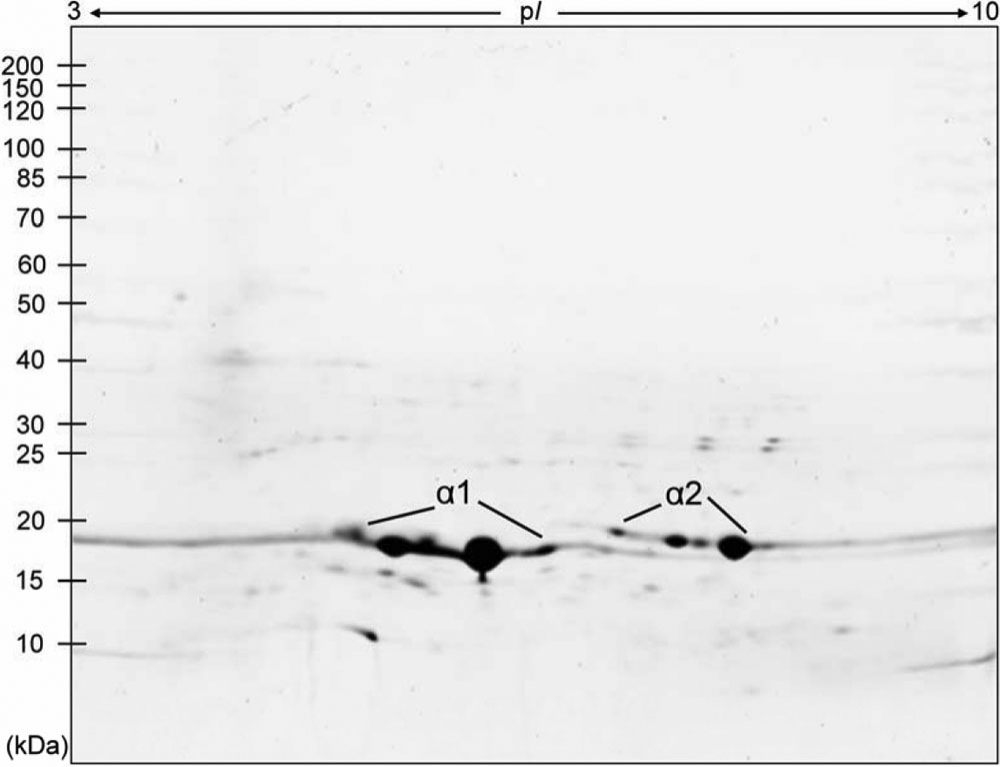
2-DE gel patterns of αA- and αB-crystallins in a porcine lens. After purification of α-crystallin by gel filtration according to our previous report [5], 100 μg α-crystallin was loaded onto IPG gel strips (pH 3–10 Nonlinear, 13 cm). The IPG strips were rehydrated, and after IEF, subjected to 2-DE. Protein spots marked and enclosed by α1 and α2 on the map denote the acidic αA-crystallin and basic αB-crystallin subunits of native α-crystallin, respectively. They were further digested by trypsin and identified by nano LC-MS/MS. It is noted that only αB-crystallin spots were found to be phosphorylated by gel-based 2-DE proteomic analysis.

**Figure 3:**
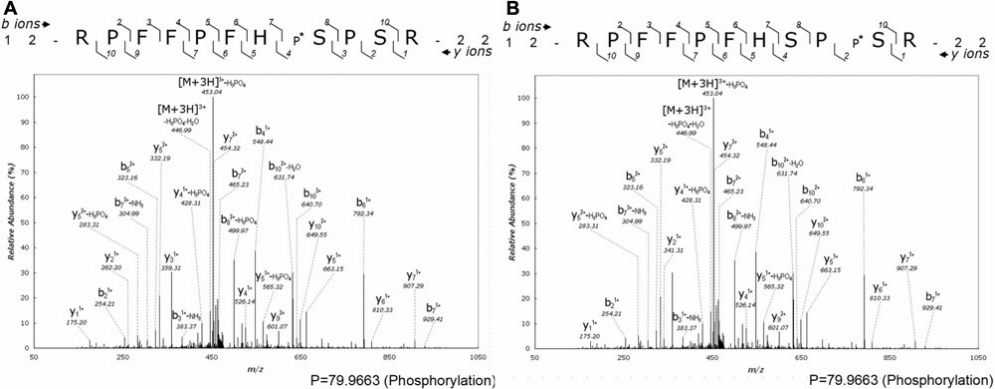
The representative tandem mass spectra of the phosphorylated peptides ^12^RPFFPFHS*PSR^22^ and ^12^RPFFPFHSPS*R^22^. **A**: MS/MS spectrum of the peptide phosphorylated at Ser-19. **B**: MS/MS spectrum of the peptide phosphorylated at Ser-21. The purified phosphopeptides samples less than 1 μg each from IMAC were first injected into a 2 cm×180 μm capillary trap column followed by LC-MS/MS and spectra collection. Based on the tandem mass spectra of the modified peptides ^12^RPFFPFHS*PSR^22^ and ^12^RPFFPFHSPS*R^22^ as compared with the original peptide, it can be deduced that either Ser-19 or Ser-21 is phosphorylated. The location of the peptide fragment within the protein is shown by the residue numbers 12 and 22 for the NH_2_- and COOH-terminus of the phosphorylated peptide sequence. Identified b- and y-ion fragment series are marked by the numbers above and under the peptide sequence, respectively. The putative site of phosphorylation is indicated by * and P* next to serine residues. The mass signals were amplified fivefold, except the ion with the highest intensity.

Because αB-crystallin is the only phosphorylated crystallin in the global protein-expression profile of total porcine lens proteins, we further confirmed the phosphorylation site of αB-crystallin by isolation and purification of α-crystallin on native gel-filtration chromatography of total lens extract [5], followed by 2-DE and LC-MS/MS analysis. As shown in Figure 2, αB-crystallin spots (marked as α2) were located in the basic region, in contrast to αA-crystallin spots (marked as α1) in the acidic side of the gel. After LC-MS/MS analysis of these protein spots, the same phosphorylated peptides ^12^RPFFPFHS*PSR^22^ and ^12^RPFFPFHSPS*R^22^ present in αB-crystallin were found (Figure 3). Therefore, we demonstrated that phosphorylation sites at Ser-19 and Ser-21 in αB-crystallin were indeed identified and confirmed by gel-based proteomic analysis.

### Gel-free proteomic analysis of phosphorylated proteins in porcine lens

Because the capability of a gel-based proteomic approach to identify phosphoproteins was limited for phosphoprotein identification, we adopted instead for a gel-free protocol similar to shotgun proteomic approaches [11,12]. By enrichment of the porcine lens phosphopeptides on IMAC followed by LC-MS/MS analysis, we have identified 195 phosphopeptides. Among the identified phosphopeptides, the proportions of phosphorylation on serine or threonine in the porcine lens were 85% and 15% (data not shown), respectively. As shown in Table 2, the 27 nondegenerate phosphopeptides belonged to six proteins in the porcine lens, including αB-crystallin, αA-crystallin, βB1-crystallin, β-enolase, heat shock protein β-1 (HSP27), and glucose-6-phosphate isomerase (GPI).

**Table 2:** Summary of identified phosphorylated proteins and phosphorylated sites in porcine lens proteins by IMAC followed by LC-MS/MS analysis.

**Protein [Accession number]**	**Fragment**	**Phosphopeptides**	**Designation**
αB-crystallin	12–22	RPFFPFHSPSR	Ser-19
[Q7M2W6]	12–22	RPFFPFHSPSR	Ser-21
	57–69	APSWIDTGLSEMR	Ser-59
	57–69	APSWIDTGLSEMR	Thr-63
	73–82	DRFSVNLDVK	Ser-76
	124–149	IPADVDPLTITSSLSSDGVLTVNGPR	Ser-139
	164–174	EEKPAVTAAPK	Thr-170
αA-crystallin	13–21	ALGPFYPSR	Ser-20
[P02475]	55–65	TVLDSGVSEVR	Ser-59
	55–65	TVLDSGVSEVR	Ser-62
	55–70	TVLDSGVSEVRSDRDK	Ser-66
	79–88	HFSPEDLTVK	Ser-81
	79–88	HFSPEDLTVK	Thr-86
	146–157	VPSGVDAGHSER	Ser-148
	146–157	VPSGVDAGHSER	Ser-155
	158–173	AIPVSREEKPSSAPTS	Ser-173
Beta-crystallin B1	8–21	ASATAAVNPGPDGK	Ser-9
[Q007T1]	8–21	ASATAAVNPGPDGK	Ser-11
	88–107	VRSIIVTSGPWVAFEQSNFR	Ser-90
	90–107	SIIVTSGPWVAFEQSNFR	Ser-95
	185–199	VSSGTWVGYQYPGYR	Thr-189
Beta-enolase	81–89	KLSVVDQEK	Ser-83
[Q1KYT0]	257–269	YDLDFKSPDDPSR	Ser-263
Heat shock protein beta-1	13–20	SPSWDPFR	Ser-13
[Q5S1U1]	13–20	SPSWDPFR	Ser-15
Glucose-6-phosphate isomerase	227–241	EWFLQSAKDPSAVAK	Ser-232
[NP_999495]	448–461	ELQAAGKSPEDFEK	Ser-455

In the “Phosphopeptides” column, the phosphorylation sites are underlined.

**Figure 4:**
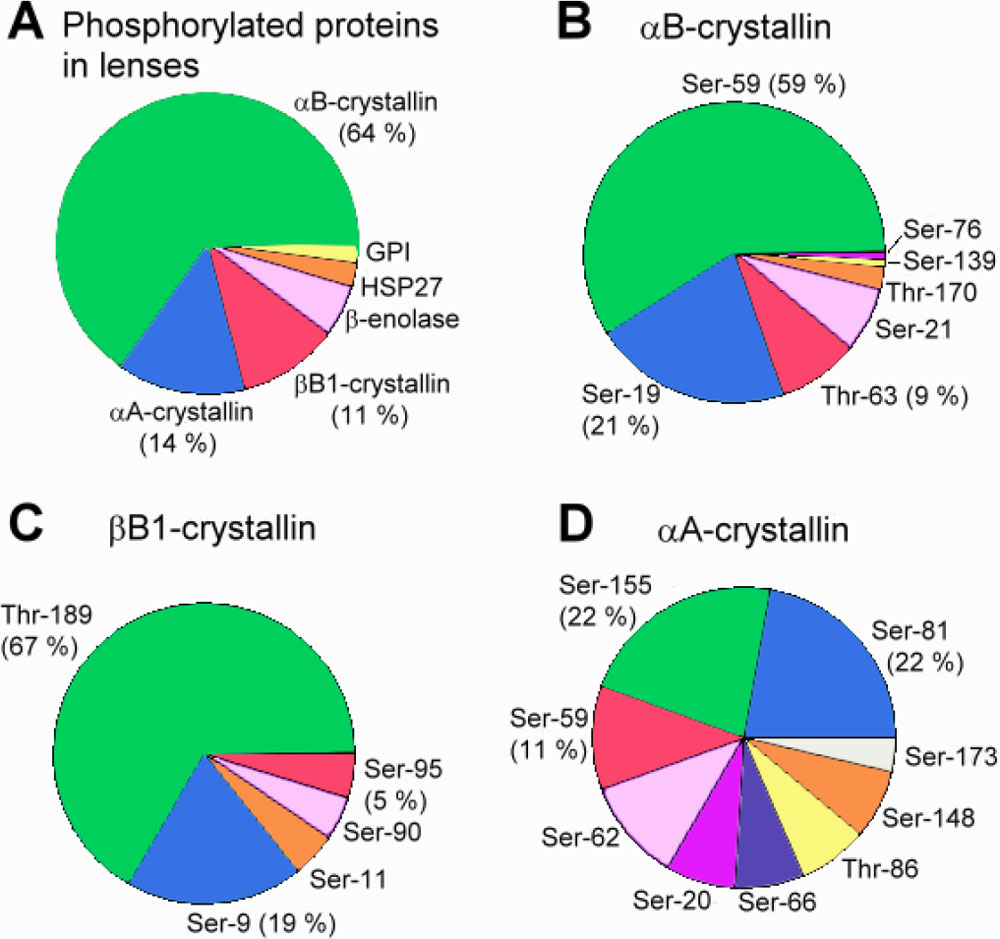
The percent distribution of phosphorylated sites identified by gel-free IMAC enriched phosphopeptide and LC-MS/MS analysis. **A**: Proportion of the proteins with phosphorylation in total lens extract. **B**: Distribution of in vivo phosphorylation sites in αB-crystallin. **C**: Distribution of in vivo phosphorylation sites in βB1-crystallin. **D**: Distribution of in vivo phosphorylation sites in αA-crystallin. The three most abundantly phosphorylated proteins (%) in the lens are shown under the identified phosphoproteins in **A**-**D**. It is noted that phosphorylated sites of αA-crystallin are more evenly distributed along the protein molecule than αB- and βB1-crystallins which show the predominant phosphorylation sites at residues 59 and 189 in αB- and βB1-crystallins, respectively.

In contrast to traditional gel-based proteomic analysis, the gel-free methods can analyze all compositions of phosphopeptides in the porcine lens. As shown in Figure 4A, most phosphopeptides were identified from αB-crystallin, indicating that it is probably the most abundant phosphoprotein in the porcine lens tissue. The proportion of other phosphopeptides identified in αA-crystallin, βB1-crystallin, β-enolase, HSP27 and GPI were 14%, 11%, 6%, 3%, and 2%, respectively, emphasizing the fact that α-crystallin consisting of αA- and αB-crystallin subunits is indeed the major phosphorylation target in the lens and may play a significant role in the phosphorylation-related biosignaling function of transparent lenses.

### Identification of phosphorylation sites in lens crystallins

As shown in Table 2, the phosphorylation sites of αA-crystallin, αB-crystallin, and βB1-crystallin were found to spread over the entire polypeptide regions of these three crystallins. Based on the proportion of phosphorylation sites in each crystallin, we found that Ser-59 and Thr-189 are two predominant phosphorylation-sites in αB-crystallin and βB1-crystallin, respectively (Figure 4B,C). To our knowledge, phosphorylation at Thr-189 of βB1-crystallin identified in this study is a new and first-reported phosphorylation site for β-crystallin class of lens crystallins. In addition, nine phosphorylated sites of αA-crystallin were found to distribute more or less evenly on the whole polypeptide chain with the exception of Ser-155, Ser-81, and Ser-59 (Figure 4D). In contrast the phosphorylation of αB-crystallin was shown to distribute unevenly over the whole crystallin with the highest proportion of phosphorylation occurring at Ser-59 followed by Ser-19 (Figure 4B). The mechanisms that account for the different extents of phosphorylation at specific sites of αA- and αB-crystallins remain unknown and is to be investigated in the future.

### Identification of phosphorylation sites in β-enolase, glucose-6-phosphate isomerase (GPI), and heat shock protein β-1 (HSP 27)

In addition to lens crystallins, three non-crystallin proteins were also found to be phosphorylated in vivo in our proteomic analysis (Table 2). The β-enolase was found to be phosphorylated at Ser-83 and Ser-263 and GPI phosphorylated at Ser-232 and Ser-455. It is noteworthy that similar to αB-crystallin, a member of the heat shock protein family, a lenticular HSP27 with chaperone activity was shown to be phosphorylated at Ser-13 and Ser-15.

### Conclusions

Besides αA- and αB-crystallins which show chaperone activity and extensive phosphorylation, βB1-crystallin and non-crystallin cellular proteins, such as β-enolase, heat shock protein β-1 (HSP27), and glucose-6-phosphate isomerase have also been shown for the first time to be phosphorylated in vivo at specific sites. Moreover, αA- and αB-crystallins were found to be the most abundantly phosphorylated proteins in porcine lenses, being exclusively phosphorylated on serine or threonine but not on tyrosine residues. Using the gel-free proteomic strategy by employing IMAC enrichment of phosphopeptides from a trypsin-digested lens protein mixture followed by sensitive LC-MS/MS proves to be superior to conventional proteomic analysis based on the pre-MS/MS 2-DE separation of protein samples. The improved strategy of gel-free phosphoproteomics analysis affords a more effective and facile method for the determination of in vivo phosphorylation sites of whole tissue extract. The use of site-directed mutagenic substitution of Asp for the phosphorylated sites of Ser or Thr residues to mimic the phosphorylation status of chaperoning αA- or αB-crystallin [27-30] will help elucidate the role of phosphorylation in relation to the chaperone-like activity and their biological significance [25,31,32].

## References

[1] Harding JJ Dilley KJ Structural proteins of the mammalian lens: a review with emphasis on changes in development, aging and cataract. Exp Eye Res 1976 22 1 73 767125 10.1016/0014-4835(76)90033-6

[2] de Jong WW Hendriks W Mulders JW Bloemendal H Evolution of eye lens crystallins: the stress connection. Trends Biochem Sci 1989 14 365 8 2688200 10.1016/0968-0004(89)90009-1

[3] Van Kleef FS De Jong WW Hoenders HJ Stepwise degradations and deamidation of the eye lens protein alpha-crystallin in ageing. Nature 1975 258 264 6 1202360 10.1038/258264a0

[4] Miesbauer LR Zhou X Yang Z Sun Y Smith DL Smith JB Post-translational modifications of water-soluble human lens crystallins from young adults. J Biol Chem 1994 269 12494 502 8175657

[5] Liao JH Lee JS Wu SH Chiou SH COOH-terminal truncations and site-directed mutations enhance thermostability and chaperone-like activity of porcine alphaB-crystallin. Mol Vis 2009 15 1429 44 19641632 PMC2716931

[6] Pawson T Scott JD Signaling through scaffold, anchoring, and adaptor proteins. Science 1997 278 2075 80 9405336 10.1126/science.278.5346.2075

[7] MacCoss MJ McDonald WH Saraf A Sadygov R Clark JM Tasto JJ Gould KL Wolters D Washburn M Weiss A Clark JI Yates JR 3rd Shotgun identification of protein modifications from protein complexes and lens tissue. Proc Natl Acad Sci USA 2002 99 7900 5 12060738 10.1073/pnas.122231399 PMC122992

[8] Chiou SH Wu SH Evaluation of commonly used electrophoretic methods for the analysis of proteins and peptides and their application to biotechnology. Anal Chim Acta 1999 383 47 60

[9] Han CL Chien CW Chen WC Chen YR Wu CP Li H Chen YJ A multiplexed quantitative strategy for membrane proteomics: opportunities for mining therapeutic targets for autosomal dominant polycystic kidney disease. Mol Cell Proteomics 2008 7 1983 97 18490355 10.1074/mcp.M800068-MCP200

[10] Ong SE Blagoev B Kratchmarova I Kristensen DB Steen H Pandey A Mann M Stable isotope labeling by amino acids in cell culture, SILAC, as a simple and accurate approach to expression proteomics. Mol Cell Proteomics 2002 1 376 86 12118079 10.1074/mcp.m200025-mcp200

[11] McCormack AL Schieltz DM Goode B Yang S Barnes G Drubin D Yates JR 3rd Direct analysis and identification of proteins in mixtures by LC/MS/MS and database searching at the low-femtomole level. Anal Chem 1997 69 767 76 9043199 10.1021/ac960799q

[12] Washburn MP Wolters D Yates JR 3rd Large-scale analysis of the yeast proteome by multidimensional protein identification technology. Nat Biotechnol 2001 19 242 7 11231557 10.1038/85686

[13] Chiou SH Azari P Himmel ME Squire PG Isolation and physical characterization of bovine lens crystallins. Int J Pept Protein Res 1979 13 409 17 457334 10.1111/j.1399-3011.1979.tb01900.x

[14] Chiou SH Chylack LT Jr Tung WH Bunn HF Nonenzymatic glycosylation of bovine lens crystallins. Effect of aging. J Biol Chem 1981 256 5176 80 7228874

[15] Liao JH Hung CC Lee JS Wu SH Chiou SH Characterization, cloning, and expression of porcine alpha B crystallin. Biochem Biophys Res Commun 1998 244 131 7 9514893 10.1006/bbrc.1998.8226

[16] Lee JS Samejima T Liao JH Wu SH Chiou SH Physiological role of the association complexes of alpha-crystallin and its substrates on the chaperone activity. Biochem Biophys Res Commun 1998 244 379 83 9514930 10.1006/bbrc.1998.8272

[17] Liao JH Lee JS Chiou SH Distinct roles of alphaA- and alphaB-crystallins under thermal and UV stresses. Biochem Biophys Res Commun 2002 295 854 61 12127973 10.1016/s0006-291x(02)00784-2

[18] Lu X Zhu H Tube-gel digestion: a novel proteomic approach for high throughput analysis of membrane proteins. Mol Cell Proteomics 2005 4 1948 58 16150870 10.1074/mcp.M500138-MCP200 PMC1360194

[19] Tsai CF Wang YT Chen YR Lai CY Lin PY Pan KT Chen JY Khoo KH Chen YJ Immobilized metal affinity chromatography revisited: pH/acid control toward high selectivity in phosphoproteomics. J Proteome Res 2008 7 4058 69 18707149 10.1021/pr800364d

[20] Ideker T Thorsson V Ranish JA Christmas R Buhler J Eng JK Bumgarner R Goodlett DR Aebersold R Hood L Integrated genomic and proteomic analyses of a systematically perturbed metabolic network. Science 2001 292 929 34 11340206 10.1126/science.292.5518.929

[21] Zhou H Watts JD Aebersold R A systematic approach to the analysis of protein phosphorylation. Nat Biotechnol 2001 19 375 8 11283598 10.1038/86777

[22] Spector A Chiesa R Sredy J Garner W cAMP-dependent phosphorylation of bovine lens alpha-crystallin. Proc Natl Acad Sci USA 1985 82 4712 6 2991889 10.1073/pnas.82.14.4712 PMC390974

[23] Chiesa R Gawinowicz-Kolks MA Kleiman NJ Spector A Definition and comparison of the phosphorylation sites of the A and B chains of bovine alpha-crystallin. Exp Eye Res 1988 46 199 208 3350065 10.1016/s0014-4835(88)80077-0

[24] Voorter CE de Haard-Hoekman WA Roersma ES Meyer HE Bloemendal H de Jong WW The in vivo phosphorylation sites of bovine alpha B-crystallin. FEBS Lett 1989 259 50 2 2599111 10.1016/0014-5793(89)81491-7

[25] Aquilina JA Benesch JL Ding LL Yaron O Horwitz J Robinson CV Phosphorylation of alphaB-crystallin alters chaperone function through loss of dimeric substructure. J Biol Chem 2004 279 28675 80 15117944 10.1074/jbc.M403348200

[26] Smith JB Thevenon-Emeric G Smith DL Green B Elucidation of the primary structures of proteins by mass spectrometry. Anal Biochem 1991 193 118 24 2042736 10.1016/0003-2697(91)90050-4

[27] Nicholl ID Quinlan RA Chaperone activity of alpha-crystallins modulates intermediate filament assembly. EMBO J 1994 13 945 53 7906647 10.1002/j.1460-2075.1994.tb06339.x PMC394896

[28] Kamei A Hamaguchi T Matsuura N Masuda K Does post-translational modification influence chaperone-like activity of alpha-crystallin? I. Study on phosphorylation. Biol Pharm Bull 2001 24 96 9 11201254 10.1248/bpb.24.96

[29] Moroni M Garland D In vitro dephosphorylation of alpha-crystallin is dependent on the state of oligomerization. Biochim Biophys Acta 2001 1546 282 90 11295434 10.1016/s0167-4838(01)00154-6

[30] Ito H Kamei K Iwamoto I Inaguma Y Nohara D Kato K Phosphorylation-induced change of the oligomerization state of alpha B-crystallin. J Biol Chem 2001 276 5346 52 11096101 10.1074/jbc.M009004200

[31] Ahmad MF Raman B Ramakrishna T Rao Ch M Effect of phosphorylation on alpha B-crystallin: differences in stability, subunit exchange and chaperone activity of homo and mixed oligomers of alpha B-crystallin and its phosphorylation-mimicking mutant. J Mol Biol 2008 375 1040 51 18061612 10.1016/j.jmb.2007.11.019

[32] Aggeli IK Beis I Gaitanaki C Oxidative stress and calpain inhibition induce alpha B-crystallin phosphorylation via p38-MAPK and calcium signalling pathways in H9c2 cells. Cell Signal 2008 20 1292 302 18420382 10.1016/j.cellsig.2008.02.019

